# Book Review: Psychiatric Diagnosis Revisited: From DSM to Clinical Case Formulation

**DOI:** 10.3389/fpsyg.2017.01182

**Published:** 2017-07-13

**Authors:** Jonathan D. Redmond

**Affiliations:** School of Counseling, The Australian College of Applied Psychology Melbourne, VIC, Australia

**Keywords:** DSM-5, DSM classification, case formulation, diagnosis, validity, reliability

The latest publication from Vanheule (2017) both critiques the DSM-5 and provides an alternative diagnostic approach for clinicians working in the “psy-disciplines” via clinical case formulation. Additionally, non-clinicians—particularly researchers, academics and the interested public—with an interest in mental health, psychiatry and the DSM diagnosis will find a highly detailed analysis of the scientific status of the DSM-5's classificatory system and historical shifts underlying different iterations of the DSM. *Psychiatric diagnosis revisited* consists of four chapters (excluding the introduction): the first two chapters critique the DSM, while the remaining two develop an alternative approach to diagnosis by providing a functional approach—as opposed to a reduction biological perspective—to symptom formations that is contextualized in the context of the clinical case formulation. It's a relatively short book of 243 pages including references; the detailed bibliography will be useful for those wishing to follow-up on specific research publications and or validate some of the many arguments that Vanheule makes throughout the text. In fact, one of the strengths of the text is that highly detailed scientific, philosophical and critical reasoning provides a sustained critique of the poor scientific reasoning and dubious procedures linked to the creation of the DSM, particularly DSM-III to DSM-5. In what follows, I outline some core areas of interest in Vanheule's book with a focus on the “bad science” underling the construction of the DSM classification system and then discuss his alternative method to diagnosis development in his clinical case formulation.

The long awaited publication of the DSM-5 (American Psychiatric Association, 2013) has been met with a high level of social and critical response by significant mental health institutions, researchers, commentators and other interest groups. This suspicion and distrust in the DSM provides a backdrop to Vanheule's work; and while a loss of faith in the DSM is socially evident—i.e. the negative impact of stigma from diagnosis, the perceived cooptation of psychiatry with the pharmacological industry, or the pathologization of normal human experiences (grief, mood variability)—what is often lacking in these discussions is a sustained critical response that engages the DSM system of classification on it's *own methodological terms*. A considerable strength of Vanheule's book is that he provides a scientific critique of the DSM. As a clinical/academic psychologist with specialized training in quantitative and qualitative research methods, psychodiagnostic assessment and statistics, Vanheule uses the concepts of reliability and validity to investigate the scientific status of the DSM-5's classificatory system. For example, chapter 1 outlines key shifts in how diagnosis was conceived across new iterations of the DSM, in particular the paradigm shift from prototype diagnosis in early editions of the DSM to a check-list/category style diagnostic system from the DSM-III onwards. This shift aimed at increasing inter-reliability for diagnosis and moved to a biologically based disease model of mental illness: he argues, by drawing on principles of reliability and validity, that neither aim has been fulfilled.

Vanheule provides a detailed discussion of reliability (specifically inter-rater reliability or the likelihood of diagnostic agreement between multiple clinicians when dealing with the same client) by focusing on the use of kappa coefficients in both the transition from prototype based diagnosis (evident in the first two volumes of the DSM) through to check-list diagnosis underlying the DSM-III onwards. His critical analysis of Kappa co-efficient norms underlying inter-rater reliability, derived from landmark studies from Spitzer and Fliess (1974), Landis and Koch (1977) and Clarke et al. (2013), highlights no substantial progress in the reliability of disorder categories from the DSM-II onwards. His conclusion: “DSM-5 is no means more statistically reliable than it was 40 years ago” (2017, pg. 3). One *simple* reason is that beyond limited field trials scientific assessment of the DSM's psychiatric disorders are absent. He states that “the majority of DSM-5 diagnostic categories were not tested at all: the DSM-5 counts 347 disorder categories, but kappa coefficients could be calculated only for 20 conditions (6%). Moreover of those categories only 14% had a good or very good reliability, which means that only 4% of the DSM-5 categories have been shown to have sufficient reliability. Indeed, since the inter-rater reliability of the majority of the DSM-5 categories remains untested, the idea that the DSM is a reliable instrument in simply wrong” (2017, pg. 62). The first 2 chapters of the book highlight the unsound scientific practices, including the poor construct validity of disorder categories, again with reference to studies from Spitzer and Fliess (1974), Landis and Koch (1977) and Clarke et al. (2013) and a series of arguments critiquing the realist epistemological assumptions that informs the disease based model of the DSM-II onwards. Moreover, when combined with other critical and epistemological perspectives—such as the problem of reification and stigma in diagnosis, the absence of biological markers underlying mental disorders and the financial links of individual's standing on the DSM-5 committee (i.e., that 69% of the DSM-5 panel members who are responsible for creating the 347 disorder categories had financial interests with the pharmacological industry)—the book achieves an impressive critique of contemporary psychiatry.

The remainder of the book provides an alternative to DSM-5 based diagnosis in the form of clinical case formulation and symptoms. These two chapters are full of detailed arguments concerning the utility of detailed context specific case formulations based on the *singular* qualities of the individual's symptoms; he outlines a function oriented diagnosis where symptoms are mapped as markers of distress articulated in speech with a unique history and meaning. In returning to the question of diagnosis via symptoms, but this time without the DSM as a reference, Vanheule focuses on a mapping of psychopathology—as opposed to abnormality and mental disorder—and does so by deconstructing what we mean by psychopathology; he does so via the philosopher Ricoeur who differentiates pain from *pathos*. Pathos refers to mental states that are: bad or undesirable; that create psychological pain and therefore and unhappiness; and, that should be treatable. The discussion of pathos is useful in highlighting how mental suffering is an intimate experience communicated through the unique expressions of the sufferer. He argues that such suffering is structured around an impasse concerning the *impossibility of transforming self-experience*. The mapping of mental suffering onto two orthogonal axes—Self/Other and Languishing/Acting—provides a useful assessment tool for identifying pathos according to a range of points of distress including social isolation, inhibitory behaviors, passivity, inability to speak about distressful experiences and the devaluation of self. This discussion builds on Vanheule's earlier discussion of symptom in chapter 2 where he develops a concept symptomatology in reference to the Cambridge Model of Symptoms (pg. 94) and his own Triangular Model of Symptom Formation (pg. 101) that de-emphasizes biological determinates of symptoms while placing greater focus on socio-cultural elements and speech acts developed using Lacan's concept of the imaginary, symbolic and real. Chapters 4 and 5 contain a great deal of information concerning how diagnosis can be approached relative to the singular status of symptoms, which in turn, is linked to the development of the clinical case formulation. There are too many details to mention here but clinicians interested in revisiting how they structure clinical consultations and approach case formulation will find it worth the time and effort.

It is worth noting that chapter 5, by revisiting the question of reliability and validity, aims to provide clinicians with a *reliable* and *valid* approach to a non-DSM based approach to diagnosis and case formulation by drawing on qualitative research methods. That is Vanheule argues that a case formulation approach to diagnosis—where symptoms forms the basis of diagnosis as opposed to syndromes or disease entities—can be reliable and valid if a series of checks and balances are integrated into the diagnosis and formulation process—i.e., reflexivity on the part of the clinician (examining assumptions, biases, impact of institutional settings on decision making), taking notes of the sessions, using a clinical diary, utilizing supervision, work discussion, personal therapy and continual engagement with the knowledge and theoretical framework of the related psy-discipline. And while many of these practices will be familiar to working clinicians, Vanheule's argues that when done consistently and reflectively, these practices provide the basis for increasing the reliability and validity of clinical case formulation.

My overall impression is that *Psychiatric diagnosis revisited* is a thought provoking book relevant to a broad range of theorists, academic and clinicians interested in both the scientific/epistemological status of DSM and to clinical practice. It provides a rational and scientifically informed critique of the DSM and provides an alternative framework of diagnosis based on careful—that is reliable and valid—practices of clinical case formulation. The text suffers from the overuse of the phrase “a plea for…” and under-explored area concerns the tension between implementing good clinical practices, which takes *considerable* time and care, with the “market driven” time pressures facing the average mental health practitioner vis-à-vis high case loads, administrative burdens and short term treatment protocols. In such contexts, implementing the full range of practices required for valid and reliable case formulation is a formidable challenge; that said, Vanheule's book makes the case for an evidence based practice where more time—for both direct clinical contact and case formulation practice—is a *necessary condition for best clinical practice*.

## Author contributions

The author confirms being the sole contributor of this work and approved it for publication.

### Conflict of interest statement

The author declares that the research was conducted in the absence of any commercial or financial relationships that could be construed as a potential conflict of interest.

## References

[B1] American Psychiatric Association (2013). Diagnostic and Statistical Manual of Mental Disorders – Fifth Edition – DSM-5. Washington, DC: American Psychiatric Association.

[B2] Clarke D. E. Narrow W. E. Regier D. A. Kuramoto S. J. Kupfer D. J. Kuhl E. A.. (2013). DSM-5 field trials in the United States and Canada, part I: study design, sampling strategy, implementation, and analytic approaches. Am. J. Psychiatry 170, 43–58. 10.1176/appi.ajp.2012.12070998 23111546

[B3] Landis J. R. Koch G. G. (1977). The measurement of observer agreement for categorical data. Biometrics 33, 159–174. 843571

[B4] Spitzer R. L. Fliess J. L. (1974). A re-analysis of the reliability of psychiatric diagnosis. British J. Psychiatry 125, 341–347. 4425771 10.1192/bjp.125.4.341

[B5] Vanheule S. (2017). Psychiatric Diagnosis Revisited: From DSM to Clinical Case Formulation. Cham: Palgrave Macmillan.

